# Solid pseudopapillary tumor of the pancreas in a 17-year-old girl

**DOI:** 10.1016/j.ijscr.2019.02.024

**Published:** 2019-02-27

**Authors:** Ayad Ahmad Mohammed, Ferhad Mohammed Rasheed, Sardar Hassan Arif, Abdulwahid M. Salih, Fahmi H. Kakamad, Shvan H. Mohammed

**Affiliations:** a University of Duhok, College of Medicine, Department of Surgery, Duhok, Kurdistan Region, Iraq; b University of Sulaimani, College of Medicine, Department of Surgery, Sulaimani, Kurdistan Region, Iraq; c Kscien Organization, Hamdi Str. Azadi Mall, Sulaimani, Kurdistan Region, Iraq; d Chara Laboratory, Shahedan Street, Kalar, Kurdistan, Iraq

**Keywords:** Pancreas, Tumor, Pseudopapillary, Pain, Solid

## Abstract

**Introduction:**

Solid pseudopapillary tumor of the pancreas is a rare tumor of low malignant potential. The aim of this paper is to present and discuss a case of solid pseudopapillary tumor of the pancreas occurring in a 17-year-old female. A 17-year-old girl presented with dull aching poorly localized left hypochondrial pain for two years, she had no clinical findings on physical examination. Ultrasound of the abdomen showed well-defined 9 cm * 7 cm heterogeneous lesion with cystic contents in the region of the tail of the pancreas. Computed tomography scan (CT scan) of the abdomen showed a mass of 8 cm * 7 cm in the region of the tail of the pancreas; that could be pancreatic mass, left suprarenal mass, or lymphoma. Resection of the mass and histopathological examination confirmed the diagnosis of pseudopapillary tumor of the pancreas.

**Conclusion:**

Pseudopapillary neoplasm of the pancreas is a rare condition, which needs surgical intervention. Close follow up is necessary to early detection of the recurrence and metastasis.

## Highlights

• Solid pseudopapillary tumor of the pancreas is a rare tumor of low malignant potential.
• It may be diagnosed incidentally, or present with nausea and vomiting.
• The aim of this paper is to present a case of solid pseudopapillary tumor of the pancreas.

## 1 Introduction

Solid pseudopapillary tumor of the pancreas is a rare tumor of low malignant potential, accounting not more than 1–2% of all exocrine tumors of the pancreas [1,2]. It is more common in females at young age. This tumor was first identified in 1959 by Virginia Frantz as a papillary cystic tumor of the pancreas, later in 1996 the tumor was defined by the World Health Organization (WHO) as “solid pseudopapillary tumors” [3,4]. It mainly affects the body and the tail of the pancreas [5]. The etiology of the tumor is unknown. It can reach to a considerable size before starting to cause symptoms [2].

The tumor may be asymptomatic and diagnosed during routine checkup. Most of the affected patients suffer from abdominal pain, abdominal discomfort, or palpable non tender upper abdominal mass. Also they may complain from nausea, vomiting, poor appetite, weight loss, or jaundice [2,4,5].

The aim of presenting this case is to raise the awareness of the surgeons to this rare tumor and clarify some of the available management options. The case was reported in line with SCARE guideline [6].

### 1.1 Patient information

A 17-year-old girl presented to the surgical department with dull aching poorly localized left hypochondrial pain for two years, the patient had no other associated symptoms. The patient had history of blunt abdominal trauma to the upper abdomen two years before presentation to which she attributed her symptom.

### 1.2 Clinical findings

The patient had no significant clinical findings on physical examination.

### 1.3 Diagnostic assessment

**Fig. 1:**
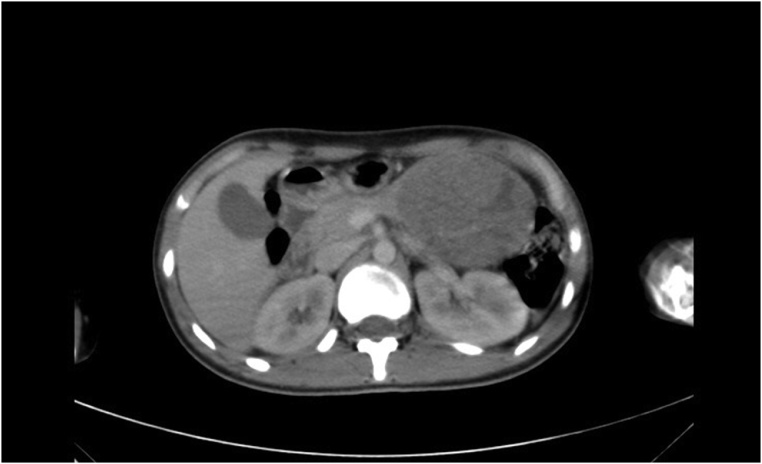
Computed tomography scan of the abdomen showing a mass in the region of the tail of the pancreas.

Ultrasound examination of the abdomen showed a well-defined 9 cm * 7 cm heterogeneous lesion with cystic contents in the region of the tail of the pancreas. Computed tomography scan (CT scan) of the abdomen showed a mass of 8 cm * 7 cm in the region of the tail of the pancreas; that could be pancreatic mass, left suprarenal mass, or lymphoma (Fig. 1). Tru-cut biopsy was performed under ultrasound guide, and histopathological examination showed features consistent with solid pseudopapillary tumor of the pancreas.

### 1.4 Therapeutic intervention

**Fig. 2:**
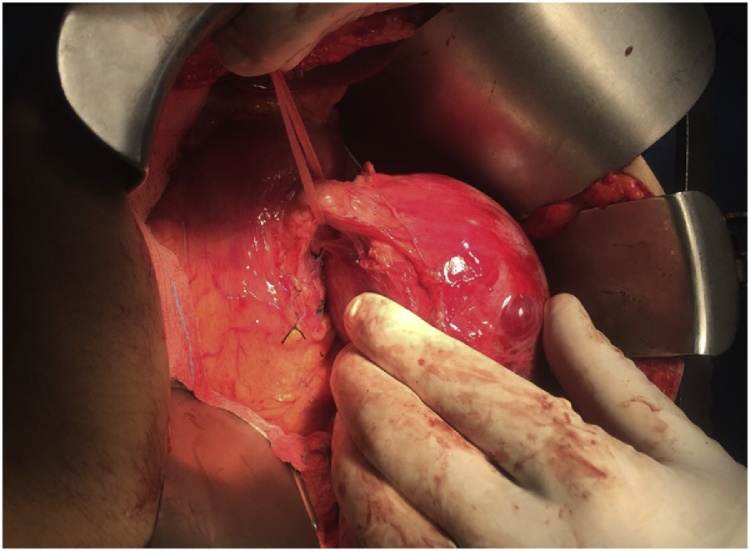
Intraoperative findings showing the pancreatic tumor dissected from the pancreatic bed. Tape is applied at the junction of the neck and body of the pancreas.

During laparotomy, after opening the lesser sac, a mass was found that was arising from the tail of the pancreas, after dissection of the mass from the pancreatic bed and the surrounding structures, distal pancreatectomy was done and specimen was sent for histopathologic examination (Fig. 2). Histopathological examination of the specimen confirmed the diagnosis of solid pseudopapillary tumor of the pancreas.

### 1.5 Follow-up and outcomes

The postoperative period passed smoothly and the patient was discharged five days after the operation.

## 2 Discussion

Diagnosis of solid pseudopapillary neoplasm of the pancreas is crucial because these tumors are distinct from other types of pancreatic cancers in that they are characterized by low malignant potential and more favorable long term outcome [7].

Characteristics for malignant tumors include, invasion of the capsule, high grade tumors, invasion of the veins, and high expression of Ki-67 on immunohistochemical analysis. The predominant strategy of management is complete surgical resection, when the tumor arises from the head of the pancreas, pancreatoduodenectomy is indicated, while if the tumor arises from the tail of the pancreas, distal pancreatectomy is sufficient [4,5]. In the current case, the tale of the pancreas was involved, that is why distal pancreatectomy including the mass was performed.

The tumor has an excellent prognosis after resection even in the presence of metastatic disease due to its favorable features, the 5-year disease free survival may reach up to 95% [2,5,7].

Large tumor size (more than 5 cm), lymphovascular invasion, metastasis to the regional lymph nodes, synchronous metastatic disease, and positive resection margins may increase the risk of tumor recurrence [1].

The tumor mainly metastasizes to the liver. Treatment of metastatic disease is resection when possible, other treatment modalities include injection of alcohol, intra-arterial embolization, or radiotherapy as these tumors are radiosensitive, even liver transplantation may be suggested by some authors [4].

Close follow up of the patients is mandatory after surgical resection for early diagnosis of local recurrence and metastatic disease [2].

In conclusion, pseudopapillary neoplasm of the pancreas is a rare condition, which needs surgical intervention. Close follow up is necessary to early detection of the recurrence and metastasis.

## Conflicts of interest

There is no conflict to be declared.

## Funding

No source to be stated.

## Ethical approval

Approval has been taken from Kscien organization for scientific research, no. 33.

## Consent

Fully informed written consent has been taken from the patient for the publication of this report.

## Author’s contribution

Ayad Ahmad Mohammed: The team leader, writing the draft with final approval of the manuscript.

Ferhad Mohammed Rasheed: revising the draft, reviewing the literature and follow up with final approval of the manuscript.

Sardar Hasan Arif: revising the draft, reviewing the literature and follow up with final approval of the manuscript.

Abdulwahid M. Salih: the surgeon who did the first operation. Final approval of the manuscript.

Fahmi Hussein Kakamad: writing the manuscript, reviewing the literature and final approval of the manuscript.

Shvan H. Mohammed: reviewing the manuscript, reviewing the literature and final approval of the manuscript.

## Registration of research studies

Not applicable.

## Guarantor

Fahmi Hussein kakamad.

## Provenance and peer review

Not commissioned, externally peer-reviewed.
